# Scrambled or flipped: 5 facts about how cellular phosphatidylserine localization can mediate viral replication

**DOI:** 10.1371/journal.ppat.1010352

**Published:** 2022-03-04

**Authors:** Marissa Danielle Acciani, Melinda Ann Brindley

**Affiliations:** 1 Department of Infectious Diseases, College of Veterinary Medicine, University of Georgia, Athens, Georgia, United States of America; 2 Department of Infectious Diseases, Department of Population Health, College of Veterinary Medicine, University of Georgia, Athens, Georgia, United States of America; University of Iowa, UNITED STATES

## Introduction

Host cell lipids are intimately involved in virus replication. Enveloped viruses are enshrouded in lipids from host cell membranes, shielding particles from the immune system and extracellular environment. Viruses can up-regulate cell lipid synthases to stimulate lipid production or recruit synthases to sites of viral replication. Furthermore, some viruses can induce autophagy to free stored lipids and optimize replication. Viruses can also remodel the composition of cell membranes, altering the concentration or localization of components such as sterols, sphingolipids, and phospholipids [1]. Here, we discuss virus-directed localization of phosphatidylserine (PtdSer), through host flippases and scramblases.

**Fig 1:**
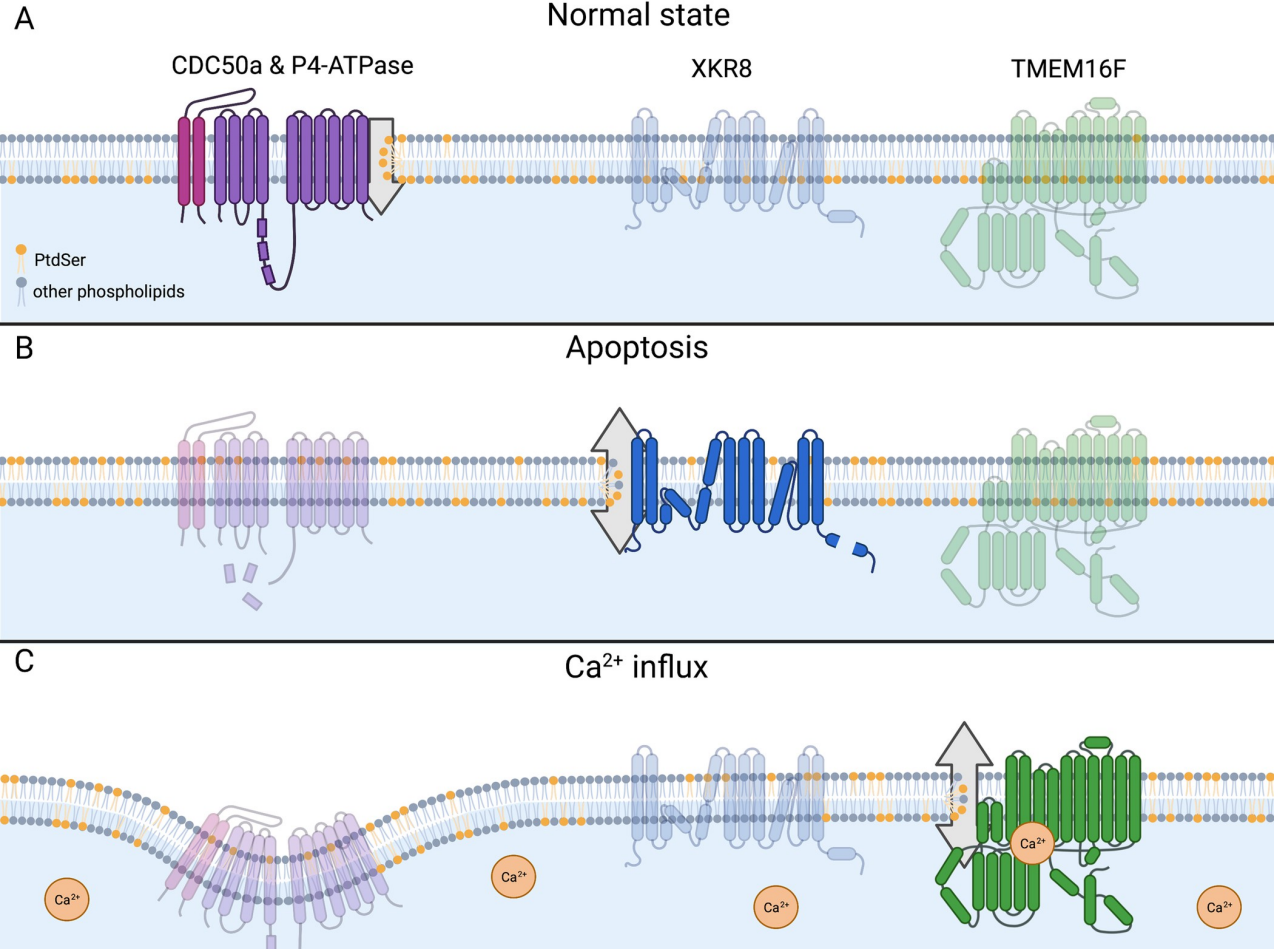
PtdSer flipping and scrambling in the plasma membrane. PtdSer is shown in orange, and all other phospholipid species are shown in gray. **(A)** In normal state cells, constitutively active flippase complexes transport outer leaflet PtdSer to the inner leaflet, resulting in highly asymmetric PtdSer distribution. **(B, C)** Flippase inactivation paired with scramblase activation results in high levels of exposed PtdSer on cell surfaces. (B) When cells undergo apoptosis, executioner caspases inactivate flippases and activate scramblase XKR8, which nonspecifically shuffles phospholipids between leaflets. (C) During calcium signaling, flippases are inactivated or down-regulated on the cell surface (mechanism is poorly characterized), while scramblase TMEM16F is activated by direct calcium binding. Like XKR8, TMEM16F nonspecifically shuffles phospholipids between leaflets. Created in BioRender.com. PtdSer, phosphatidylserine; TMEM16F, transmembrane protein 16F; XKR, XK related.

PtdSer is an essential anionic phospholipid nonuniformly distributed throughout the cell. PtdSer synthases located in the mitochondria-associated membranes of the endoplasmic reticulum (ER) produce PtdSer, which is then transported primarily to the plasma membrane via vesicular trafficking. PtdSer distribution in the plasma membrane is asymmetric, as it is strictly found in the inner/cytoplasmic leaflet. While PtdSer makes up about 15 mol% of the total lipid in the plasma membrane, it accounts for about 30 mol% of the inner leaflet. Due to its negative charge, the concentration of inner leaflet PtdSer can impact the localization of plasma membrane-associating proteins, including those required for endocytosis, and is important for promoting membrane curvature [2]. Cellular enzymes termed flippases maintain the asymmetric distribution of PtdSer (Fig 1A). Plasma membrane flippase complexes include a Type 4 P-Type ATPase (P4-ATPase) family member, which binds and flips the phospholipids, and a cell division cycle protein 50 (CDC50) family member that facilitates folding and localization of the complex [2,3].

Cells expose PtdSer during specific cell signaling events; platelet PtdSer exposure regulates blood coagulation, and apoptotic cell PtdSer exposure marks the cell for phagocytic uptake and clearance. Trans-bilayer movement of PtdSer, however, requires overcoming a high-energy barrier, with spontaneous translocation occurring at a very low rate [4]. To induce rapid PtdSer exposure, cells must inactivate flippases and activate scramblases to overcome the unfavorable action of translocating the polar head-groups across the hydrophobic bilayer (Fig 1B and 1C). Scramblases facilitate the trans-bilayer movement of phospholipids, enabling PtdSer exposure to occur rapidly [5]. Several plasma membrane-localized scramblase families have been identified. The transmembrane protein 16 (TMEM16) family includes calcium-dependent scramblases that facilitate temporary PtdSer exposure in response to an increase in intracellular calcium (Fig 1C) [6]. The XK-related (XKR) protein family includes scramblases activated by caspase cleavage. XKR8, the most well-characterized member, facilitates the nonreversible exposure of PtdSer after apoptosis induction (Fig 1B) [7]. Inside the cell, transmembrane protein 41B (TMEM41B) and vacuole membrane protein 1 (VMP1) were recently identified as an ER-localized scramblase that help distribute PtdSer in the ER membrane [8–10]. When incorporated into liposomes, these scramblases can shuffle phospholipids in an ATP- and calcium- independent manner.

In this review, we will briefly summarize how viruses utilize PtdSer-regulating enzymes during their replication cycle. While the majority of these studies investigated virus-induced PtdSer scrambling, we will also discuss several instances where flippases were linked to virus replication.

### Entry through apoptotic mimicry

**Fig 2:**
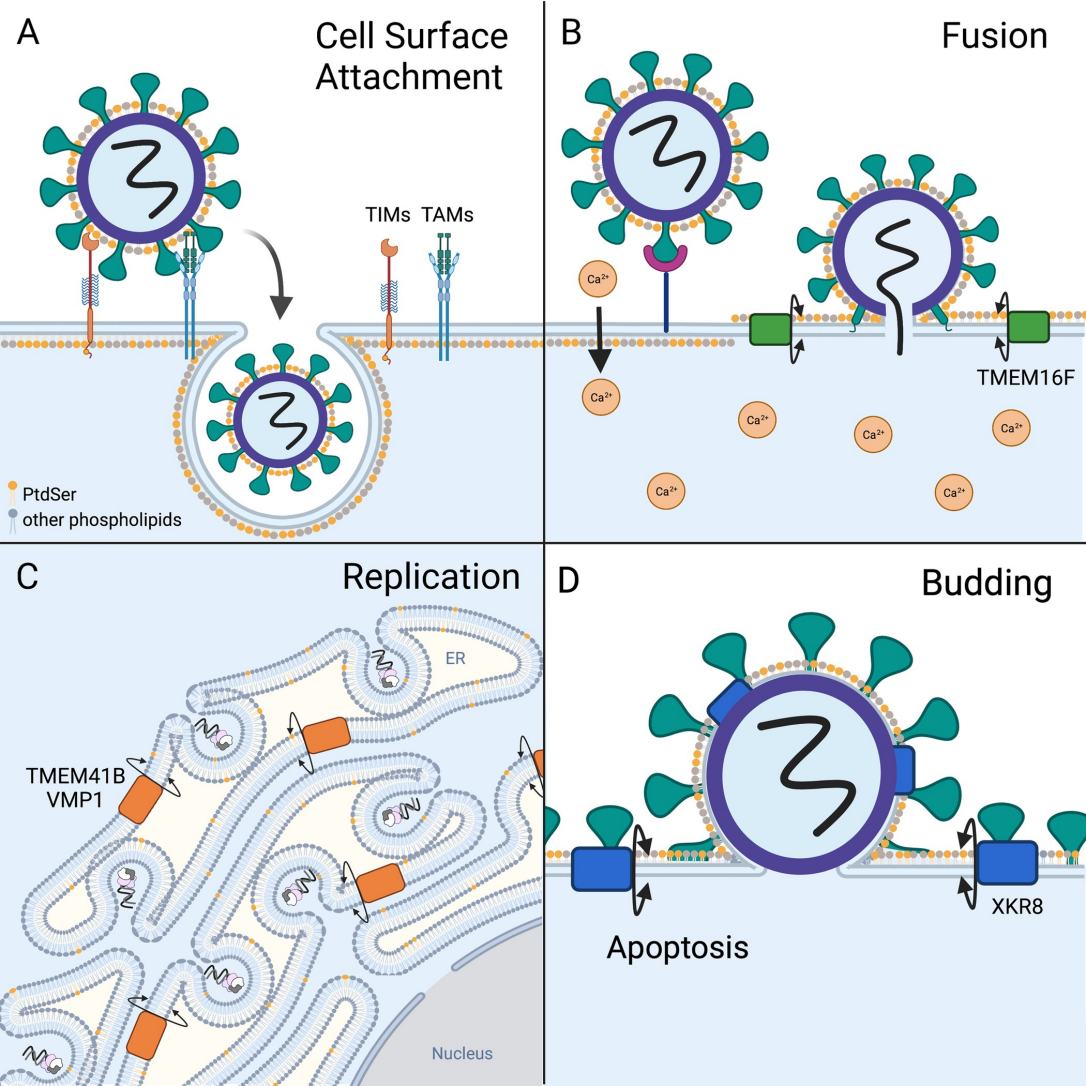
PtdSer scrambling and virus replication. PtdSer is shown in orange and all other phospholipid species are shown in gray. **(A)** Apoptotic mimicry. Enveloped viruses containing exposed PtdSer can enter cells by attaching to cell surface apoptotic clearance receptors like TIMs, TAMs, CD300 receptors, scavenger receptors, and integrins, which typically bind and internalize PtdSer-coated apoptotic debris. **(B)** Enveloped viruses that fuse at the plasma membrane, including HIV, EHV-1, and SARS-CoV-2, trigger calcium-induced PtdSer scrambling upon cell surface binding, which enhances envelope-membrane fusion. TMEM16F was specifically shown to mediate this process for HIV and SARS-CoV-2. **(C)** (+) RNA viruses such as flaviviruses and coronaviruses rely on PtdSer scramblase TMEM41B for the formation of membranous ER RC, which are the primary sites of genome replication. **(D)** PtdSer scrambling mediated by caspase-cleaved XKR8 was shown to enhance VSV and EBOV budding. It was also shown that XKR8 is incorporated into EBOV-like particle envelopes. Created in BioRender.com. EBOV, Ebola virus; EHV-1, equine herpesvirus 1; ER, endoplasmic reticulum; HIV, human immunodeficiency virus; PtdSer, phosphatidylserine; RC, replication complex; SARS-CoV-2, Severe Acute Respiratory Syndrome Coronavirus 2; TAM, Tyro3, Axl, and Mer; TIM, T-cell immunoglobulin and mucin domain; TMEM16F, transmembrane protein 16F; TMEM41B, transmembrane protein 41B; VMP1, vacuole membrane protein 1; VSV, vesicular stomatitis virus; XKR, XK related.

Numerous enveloped viruses including dengue virus [11,12], Ebola virus (EBOV) [11,13–18], chikungunya virus [11,13,19], vaccinia virus [20–22], and others [11,13,19,23–33] utilize PtdSer receptors for initial host cell attachment, a mechanism termed apoptotic mimicry (Fig 2A). PtdSer within the outer leaflet of the viral envelope mediates binding to PtdSer receptors on phagocytic cells that typically recognize PtdSer-coated apoptotic bodies. Even the infectivities of nonenveloped enteroviruses and Hepatitis A virus are enhanced through PtdSer receptors because naked particles and RNA can be delivered into cells as cargo in PtdSer-rich exosomes [27,28]. Host cells produce many receptors that bind to PtdSer including T-cell immunoglobulin and mucin domain (TIM) proteins, Tyro3, Axl, and Mer (TAM), CD300 receptors, scavenger receptors, and integrins, which can be used by viruses with some redundancy [18,34]. PtdSer receptor utilization and virus entry have recently been reviewed [35].

Viral envelope PtdSer is acquired during budding from either PtdSer-rich inner membranes or the plasma membrane. For viruses that bud from the plasma membrane, they must first alter the asymmetric PtdSer distribution to later engage PtdSer receptors. Many viruses induce apoptosis, thereby inactivating flippases and activating scramblases, increasing PtdSer on the plasma membrane and viral envelope. Some viruses also modulate cellular calcium signaling pathways to increase cytosolic calcium levels, which would trigger calcium-activated scramblases [36]. Studies examining EBOV PtdSer levels have implicated both apoptotic scramblase XKR8 [37,38] and calcium-activated scramblase TMEM16F [39] in EBOV envelope PtdSer and infectivity.

### Scramblase TMEM16F and fusion

Enveloped viruses must mediate fusion of viral and cellular membranes to initiate replication, and exposed PtdSer plays a role in this process (Fig 2B). Ptdser scrambling can alter the fusogenic properties of lipid bilayers either directly, as scrambled PtdSer can promote the formation of fusion intermediates, or indirectly by recruiting fusion machinery or restructuring fusion proteins [40].

Several studies suggest that PtdSer scrambling enhances viral fusion at the plasma membrane. For alphaherpesviruses equine herpesvirus 1 (EHV-1) and herpes simplex viruses 1 and 2 (HSV-1 and HSV-2), virus cell surface binding induced calcium signaling and PtdSer exposure, which enhanced viral fusion and entry [41,42]. Human immunodeficiency virus (HIV) and Severe Acute Respiratory Syndrome Coronavirus 2 (SARS-CoV-2), which can also fuse at the plasma membrane, activate TMEM16F scrambling during the fusion process [43–45]. TMEM16F is localized in the plasma membrane and is activated during periods of high intracellular calcium [6]. Loss of TMEM16F at the plasma membrane, or functional inhibition of TMEM16F activity with small molecules, results in failure to produce fusion pores and therefore inhibits infection and/or syncytia formation [43,44].

### Scramblases TMEM41B and VMP1 in (+)RNA viral replication

During replication, positive-sense (+)RNA viruses remodel internal cellular membranes to form replication complexes (RCs), which is thought to improve replication efficiency and shield these sites from host antiviral defenses [46]. Recent evidence suggests that PtdSer scramblases are important for (+)RNA virus replication, likely due to their impact on RC formation (Fig 2C). Independent genome-wide screens for viral host factors identified ER scramblase TMEM41B as an essential component in flavivirus and coronavirus replication [47–51]. Cells deficient in TMEM41B failed to form flavivirus RC, while other viruses that do not replicate in ER RCs were unaffected [50]. Eliminating TMEM41B in mice also significantly delayed murine coronavirus disease progression in vivo [51]. ER scramblase VMP1, which shares a number of functional and structural similarities with TMEM41B [52], also appeared in 2 of these viral host factor screens [47,50]. Cells lacking VMP1 were significantly resistant to flavivirus infection [50] and somewhat resistant to coronavirus infection [47,51]; however, in-depth mechanistic studies on how VMP1 interferes with virus replication were not performed. Likely, the loss of trans-bilayer phospholipid movement in the ER, combined with altered lipid mobilization and cholesterol distribution observed in TMEM41B and VMP1 knockout cells [9,49,50], prevents ER membrane rearrangements that enable robust genome replication of (+)RNA viruses that replicate in this compartment.

### Scramblase XKR8 and viral budding

While investigating the role of scramblase XKR8 in EBOV entry through apoptotic mimicry, our group observed that XKR8-knockout human haploid HAP1 cells consistently released fewer recombinant vesicular stomatitis virus particles and EBOV virus-like particles (VLPs) [38]. VLP release was restored by transfecting XKR8-knockout HAP1 cells with exogenous XKR8. Our data further indicated that during VLP production, caspase-activated XKR8 scrambles PtdSer in the plasma membrane, resulting in enhanced particle release (Fig 2D). Because PtdSer can induce membrane curvature, it is likely that PtdSer scrambling in the plasma membrane promotes the egress of enveloped viruses. Curiously, EBOV matrix protein VP40 must first bind to inner leaflet PtdSer during particle assembly, followed by PtdSer exposure [53]. Thus, it will be interesting to examine the exact stimuli leading to scramblase activation and how this timing is achieved to enhance particle budding. Previous work by Nanbo and colleagues did not describe altered VLP production in XKR8 knockdown HEK293T cells, suggesting that either (1) minimal levels of XKR8 can support VLP budding and/or (2) XKR8 enhances VLP budding in a cell type–dependent manner [37]. These possibilities must also be explored to fully characterize the role of Ptdser scrambling in enveloped virus budding.

### The curious case of flippases

Few studies have investigated the relationship between viruses and flippases; however, flippase subunit CDC50a has appeared in several viral host factor screens, indicating that this protein may be involved in virus entry or replication. CRISPR screens for Lujo virus and SARS-CoV-2 host cell factors identified CDC50a in HAP1 cells and Huh7 cells, respectively [48,54]. Deleting this protein in either cell type reduced cell susceptibility to virus. In determining the human-Nipah virus protein–protein interactome, CDC50a was found to interact with the antiviral Nipah virus C protein (an innate immunity antagonist) [55]. While none of these studies pursued investigations into CDC50a, this subunit may broadly affect virus replication.

Meanwhile, a study in *Arabidopsis thaliana* antiviral defense found that flippases were required for cucumber mosaic virus (CMV) resistance [56]. They found that deleting either ALA-1 or ALA-2, both P4-ATPases, increased viral load in leaves and reduced the production of antiviral and endogenous virus-activated siRNAs. The exact mechanism by which flippases support siRNA production in *A*. *thaliana*, however, remains a mystery.

## Future directions

Viruses induce an extraordinary number of changes in the host cell to transform it into an optimal virus factory. As we continue to examine the relationship between viruses and cellular lipids in particular, many questions remain.

Several of the studies addressed in this review indicate there may be a pattern between PtdSer-distributing enzymes and distinct aspects of viral replication, i.e., TMEM16F scrambling and enveloped virus plasma membrane fusion, XKR8 scrambling and enveloped virus plasma membrane budding, and TMEM41B/VMP1 scrambling and viruses that replicate in ER RCs. Thus, future studies involving these host cell factors should include viruses from additional families sharing similar replication strategies. For example, TMEM41B may also play a role in enterovirus replication in the ER. Togaviruses or nodaviruses, which replicate on endosomal and mitochondrial membranes, may employ flippases or scramblases to reorganize membrane phospholipids and form RCs [46]. Depending on how broadly essential these host factors are to virus replication, they may make promising targets for antivirals.

Flippases and scramblases may also indirectly affect viral host-protein recruitment, as enriched anionic PtdSer in membrane leaflets can promote the localization of charged-based proteins. For example, cationic stretches in protein kinase src and RasGTPase family members target these proteins to the inner leaflet of the PM in steady-state cells. PtdSer is also critical for cavin1-plasma membrane binding and caveolae formation in mammalian cells and cell division cycle protein 42 (CDC42) localization and budding in yeast [2]. Therefore, it would likely be fruitful to investigate the relationship between PtdSer localization and viral host factor recruitment particularly during the replication stages that occur at the plasma membrane.

Targeting scramblases and flippases to limit viral replication in a host may not be viable; these enzymes and the processes they mediate are highly conserved in eukaryotes and are often essential. While many groups cited in this review established viable scramblase and flippase knockout cell lines, defects in several of these proteins have been linked to diseases or disorders in mammals [8,51,57–64]. In the quest for new and broadly inhibiting antivirals, some have opted to target PtdSer enriched on viral envelopes rather than interfere with essential PtdSer scramblases. Treatments of PtdSer-binding antibodies PGN401 (Bavituximab) or PGN632 were well tolerated in guinea pigs and protected 50% of animals from fatal Pichinde virus disease (similar to Lassa virus fever in humans) alone [65] or combined with ribavirin [66]. Furthermore, PGN401 treatment protected 100% of mice from fatal murine cytomegalovirus (CMV) infection, demonstrating the broad application of the approach. These groups proposed that PGN401/PGN632 cause opsonization and clearance of viral particles from the blood and induce antibody-dependent cellular cytotoxity of virus-infected cells. Notably, the first group to examine a PtdSer-binding antibody as an antiviral based their rationale on the observations that many viruses activate PtdSer scrambling in host cells, exemplifying how elucidating the relationship between virus replication and cellular PtdSer distribution can reveal new avenues of antiviral development [65].

Furthermore, several studies reviewed here illuminate potential new tools that may be useful for improving upon current and future vaccines. The groups investigating EBOV apoptotic mimicry manipulated (specifically reduced) viral envelope PtdSer distribution and infectivity in vitro by inhibiting cellular XKR8 or TMEM16F PtdSer scrambling activity [37–39]. Biochemical studies also show that basal and apoptotic surface PtdSer levels can be elevated by overexpressing TMEM16F and XKR8 [67–69]. Therefore, it may be possible to fine-tune virus- or VLP-based vaccines by enriching or depleting exposed PtdSer. It would be interesting to examine whether vaccine PtdSer levels influence the activation of cell-mediated versus humoral immune responses, as elevated PtdSer levels may enhance vaccine uptake into antigen-presenting cells, while low PtdSer levels may promote vaccine circulation and recognition by B cells. An additional question in such experiments would be whether altering vaccine PtdSer levels impacts immunogenicity, as phagocytic uptake of PtdSer-coated bodies can dampen inflammation [70].

Considering the few flippases and scramblases discussed above have only recently been identified, there is much left to discover about the significance of these enzymes in virus replication and even cell biology in general. Their structures, functions, and upstream effectors are not yet fully characterized but will likely be critical to fully understanding how viral infection changes the lipid landscape in host cell membranes and may expose a wide array of novel targets for antivirals.
